# First‐line nivolumab, paclitaxel, carboplatin, and bevacizumab for advanced non‐squamous non‐small cell lung cancer: Updated survival analysis of the ONO‐4538‐52/TASUKI‐52 randomized controlled trial

**DOI:** 10.1002/cam4.6348

**Published:** 2023-08-28

**Authors:** Hye Ryun Kim, Shunichi Sugawara, Jong‐Seok Lee, Jin‐Hyoung Kang, Naoki Inui, Toyoaki Hida, Ki Hyeong Lee, Tatsuya Yoshida, Hiroshi Tanaka, Cheng‐Ta Yang, Makoto Nishio, Yuichiro Ohe, Tomohide Tamura, Nobuyuki Yamamoto, Chong‐Jen Yu, Hiroaki Akamatsu, Shigeru Takahashi, Kazuhiko Nakagawa

**Affiliations:** ^1^ Division of Medical Oncology, Department of Internal Medicine, Yonsei Cancer Center Yonsei University College of Medicine Seoul South Korea; ^2^ Department of Pulmonary Medicine Sendai Kousei Hospital Miyagi Japan; ^3^ Division of Hematology and Medical Oncology, Department of Internal Medicine, Seoul National University Bundang Hospital Gyeonggi‐do South Korea; ^4^ Department of Medical Oncology The Catholic University of Korea Seoul St. Mary's Hospital Seoul South Korea; ^5^ Department of Pulmonary Medicine Hamamatsu University Hospital Shizuoka Japan; ^6^ Department of Thoracic Oncology Aichi Cancer Center Aichi Japan; ^7^ Department of Internal Medicine Chungbuk National University Hospital Chungcheongbuk‐do South Korea; ^8^ Department of Thoracic Oncology National Cancer Center Hospital Tokyo Japan; ^9^ Department of Internal Medicine Niigata Cancer Center Hospital Niigata Japan; ^10^ Department of Thoracic Medicine Chang Gung Memorial Hospital Taoyuan Taiwan; ^11^ Department of Thoracic Medical Oncology Cancer Institute Hospital of Japanese Foundation for Cancer Research (JFCR) Tokyo Japan; ^12^ Thoracic Center St. Luke's International Hospital Tokyo Japan; ^13^ Internal Medicine III Wakayama Medical University Wakayama Japan; ^14^ Department of Internal Medicine National Taiwan University Hospital Hsin‐Chu Branch Hsinchu County, Taipei Taiwan; ^15^ Oncology Clinical Development Planning 1 Ono Pharmaceutical Co., Ltd. Osaka Japan; ^16^ Department of Medical Oncology Kindai University Faculty of Medicine Osaka Japan

**Keywords:** bevacizumab, chemotherapy, nivolumab, non‐squamous non‐small cell lung cancer, survival

## Abstract

**Background:**

ONO‐4538‐52/TASUKI‐52 was performed in Japan, Korea, and Taiwan to determine the oncological effectiveness and safety of combining nivolumab or placebo with bevacizumab plus platinum chemotherapy for the initial (first‐line) treatment of patients with advanced non‐squamous non‐small cell lung cancer (nsNSCLC). At the interim analysis (minimum follow‐up, 7.4 months), the independent radiology review committee‐assessed progression‐free survival was significantly longer in the nivolumab arm, but overall survival (OS) data were immature.

**Methods:**

Here, we present the updated OS data. Patients with treatment‐naïve stage IIIB/IV or recurrent nsNSCLC without driver mutations in *ALK*, *EGFR*, or *ROS1*, were randomized 1:1 to receive either nivolumab or placebo. Patients in both arms received paclitaxel, carboplatin, and bevacizumab, administered 3‐weekly for a maximum of 6 cycles. Nivolumab/placebo and bevacizumab were subsequently continued until disease progression or unacceptable toxicity.

**Results:**

Overall, 550 patients were randomized. At the time of the analysis (minimum follow‐up: 19.4 months), the median OS was longer in the nivolumab arm than in the placebo arm (30.8 vs. 24.7 months; hazard ratio 0.74, 95% confidence interval 0.58–0.94). The 12‐month OS rates were 81.3% vs. 76.3% in the nivolumab vs. placebo arms, respectively. The respective 18‐month OS rates were 69.0% vs. 61.9%.

**Conclusion:**

Nivolumab plus platinum chemotherapy and bevacizumab demonstrated longer OS vs. the placebo combination. We believe this regimen is viable as a standard, first‐line treatment for patients with advanced nsNSCLC without driver mutations in *ALK*, *EGFR*, or *ROS1*.

## Graphical abstract

The ONO‐4538‐52/TASUKI‐52 study investigated the efficacy and safety of nivolumab versus placebo, both combined with platinum chemotherapy and bevacizumab, as first‐line treatment for advanced non‐squamous non‐small cell lung cancer (NSCLC). After a minimum follow‐up of 19.4 months, overall survival significantly favored the nivolumab arm, and this survival benefit was observed regardless of programmed death‐ligand 1 expression level. These results indicate that nivolumab plus platinum chemotherapy and bevacizumab is an effective option for treatment‐naive patients with advanced non‐squamous NSCLC without driver mutations.

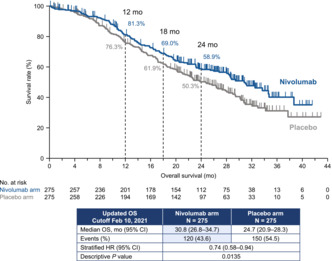

## 1 INTRODUCTION

Clinical trials have demonstrated that immune checkpoint inhibitors, when administered as monotherapy or administered together with appropriate chemotherapy regimens, are promising as first‐line treatments for non‐small cell lung cancer (NSCLC) in the absence of driver mutations^1^, ^2^; those regimens have since become the standard of care. Owing to the immunologic effects of chemotherapy, combining a chemotherapy regimen with an immune checkpoint inhibitor targeting programmed cell death‐1 (PD‐1) is expected to have synergistic antitumor effects.^2^ ONO‐4538‐52/TASUKI‐52^3^ therefore investigated the oncological effectiveness and safety of administering nivolumab (anti–PD‐1 immunotherapy) together with bevacizumab and platinum chemotherapy for the first‐line treatment of patients with advanced non‐squamous NSCLC (nsNSCLC). Upon randomization, patients received either nivolumab or placebo. Results of the interim analysis with a minimum follow‐up of 7.4 months supported the approval of nivolumab plus paclitaxel, carboplatin and bevacizumab (PCB) in Japan, Korea, and Taiwan as an initial (first‐line) treatment of metastatic nsNSCLC. Due to immature overall survival (OS) data, we performed an updated analysis of OS data with a longer minimum follow‐up (19.4 months).

In a Japanese phase II study, nivolumab numerically prolonged OS in the order of tumor programmed death‐ligand 1 (PD‐L1) expression levels of <1%, 1%–49%, and ≥ 50% in previously treated patients with non‐squamous NSCLC.^4^ On the other hand, in the ONO‐4538‐52/TASUKI‐52 study, the PFS benefit was also observed even at tumor PD‐L1 expression levels of <1% or indeterminate.^3^ Therefore, we also analyzed OS in patients divided according to their tumor PD‐L1 expression.

## 2 MATERIAL AND METHODS

As previously described,^3^ ONO‐4538‐52/TASUKI‐52 was a randomized, placebo‐controlled, double‐blind, Phase III study involving 135 study sites in three countries in Asia (Japan, Korea, and Taiwan). The study was registered as NCT03117049 (ClinicalTrials.gov) and JapicCTI‐173560 (Japan Pharmaceutical Information Center database). The study was approved by the relevant Institutional Review Board or Independent Ethics Committee at each participating site, and adhered to relevant guidelines for clinical trials (including the Declaration of Helsinki).

The eligibility criteria are described in detail in our previous report.^3^ Briefly, patients aged ≥20 years were eligible for this study if they had stage IIIB/IV or recurrent nsNSCLC, a performance status (Eastern Cooperative Oncology Group PS) of 0 or 1, and ≥1 measurable lesion (defined according to RECIST version 1.1),^5^ providing they had not previously undergone a systemic therapy for metastatic disease, were considered unsuitable for definitive radiation therapy, and had no sensitizing alterations in *ALK*, *EGFR*, or *ROS1*.

Overall, 550 patients (275 per arm)^3^ were randomized to the nivolumab arm (360 mg) or the placebo arm. Patients in both treatment arms were also treated with paclitaxel (at a dose of 200 mg/m^2^), carboplatin (at an area under the curve of 6), and bevacizumab (at a dose of 15 mg/kg) every 3 weeks for a maximum of six treatment cycles. After completing this treatment regimen, the patients were to continue nivolumab or placebo (as randomized) and bevacizumab until any of the following occurred: progressive disease (RECIST version 1.1),^5^ unacceptable toxicity, or withdrawal of consent.^3^ The randomization included stratification by sex (male vs. female), PS (0 vs. 1), and the tumor PD‐L1 expression level (≥50% vs. 1%–49% vs. <1%/indeterminate).^3^

Only data for OS were analyzed through to the cutoff date of February 10, 2021 (minimum follow‐up, 19.4 months) using the intention‐to‐treat analysis set. We used the Kaplan–Meier analysis method to estimate the OS and the Brookmeyer–Crowley method with double‐log transformation was used to calculate 95% confidence intervals (CIs). For subgroup analyses, the Cox proportional hazards model was utilized to calculate unstratified HRs (together with the 95% CIs).

## 3 RESULTS

**FIGURE 1:**
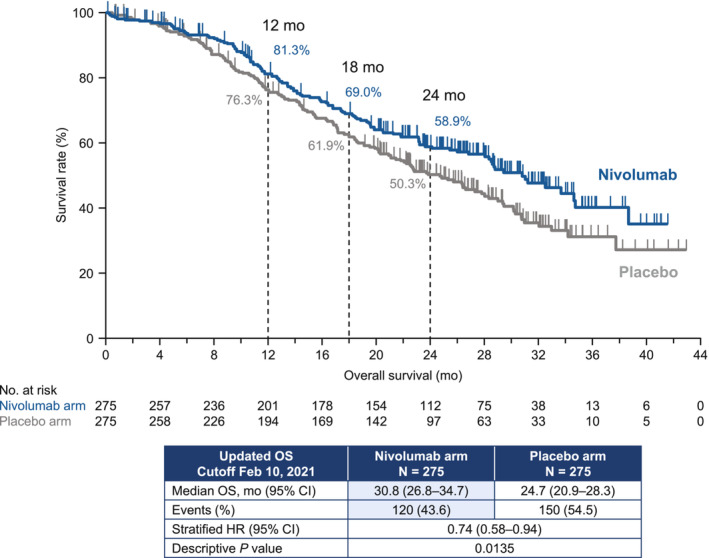
Overall survival with a minimum follow‐up period of 19.4 months (Kaplan–Meier plot). CI, confidence interval; HR, hazard ratio; OS, overall survival; mo, months.

**FIGURE 2:**
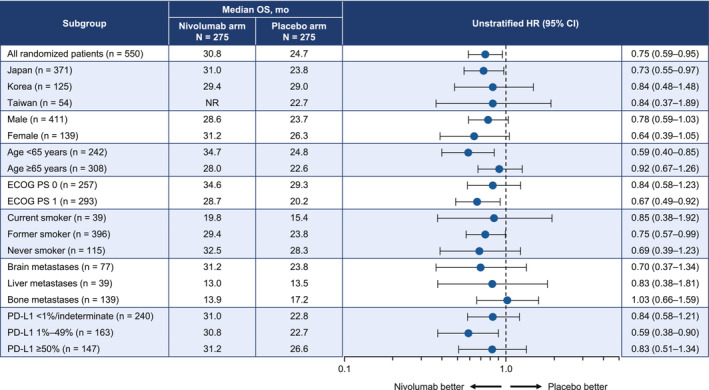
Subgroup analysis of overall survival. CI, confidence interval; ECOG PS, Eastern Cooperative Oncology Group performance status; HR, hazard ratio; mo, months; NR, not reached; OS, overall survival; PD‐L1, programmed death‐ligand 1.

**FIGURE 3:**
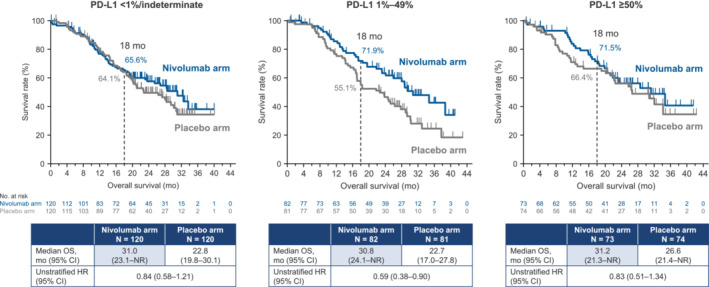
Overall survival in patients stratified by programmed death‐ligand 1 expression level (Kaplan–Meier plots). There were five patients with indeterminate PD‐L1 expression levels in both arms. CI, confidence interval; HR, hazard ratio; mo, months; OS, overall survival; PD‐L1, programmed death‐ligand 1; NR, not reached.

The baseline characteristics of patients in the nivolumab and placebo arms are presented in Table 1.^3^ A total of 120 of 275 (43.6%) events in the nivolumab arm and 150 of 275 (54.5%) events in the placebo arm were observed by the data cutoff. The median OS was 30.8 (95% CI 26.8–34.7) months at the data cutoff for patients in the nivolumab arm compared with 24.7 (95% CI 20.9–28.3) months for patients allocated to placebo (Figure 1). The HR was 0.74 (95% CI 0.58–0.94), which corresponds to a 26% lower risk of death for nivolumab relative to placebo. The estimated OS was 81.3% in the nivolumab arm vs. 76.3% in the placebo arm at 12 months, with corresponding values of 69.0% and 61.9% at 18 months. In the subgroup analyses, there were consistently better OS rates in the nivolumab arm, except in patients with bone metastasis (Figure 2). When patients were divided into three categories according to tumor expression levels of PD‐L1, the nivolumab arm had a numerically longer median OS for each category vs. the placebo arm (<1%/indeterminate: 31.0 vs. 22.8 months; 1%–49%: 30.8 vs. 22.7 months; ≥50%: 31.2 vs. 26.6 months; Figure 3). In these three subgroups, the HRs (95% CI) were 0.84 (95% CI 0.58–1.21), 0.59 (95% CI 0.38–0.90), and 0.83 (95% CI 0.51–1.34), respectively.

**TABLE 1:** Baseline patient characteristics.

Characteristic	Nivolumab arm (*N* = 275)	Placebo arm (*N* = 275)
Age (years)		
Median	66	66
Range	27–85	33–83
Age group, *n* (%)		
<65 years	131 (47.6)	111 (40.4)
65–74 years	117 (42.5)	131 (47.6)
≥75 years	27 (9.8)	33 (12.0)
Sex^a^, *n* (%)		
Male	205 (74.5)	206 (74.9)
Female	70 (25.5)	69 (25.1)
Country, *n* (%)		
Japan	188 (68.4)	183 (66.5)
Korea	62 (22.5)	63 (22.9)
Taiwan	25 (9.1)	29 (10.5)
ECOG PS score^a^, *n* (%)		
0	129 (46.9)	128 (46.5)
1	146 (53.1)	147 (53.5)
Smoking status^b^, *n* (%)		
Never	61 (22.2)	54 (19.6)
Current	18 (6.5)	21 (7.6)
Former	196 (71.3)	200 (72.7)
Staging (UICC‐TNM classification, 7th edition), *n* (%)		
Stage IIIB	15 (5.5)	14 (5.1)
Stage IV	239 (86.9)	238 (86.5)
Recurrent	21 (7.6)	23 (8.4)
Metastasis, *n* (%)		
Bone	56 (20.4)	83 (30.2)
Liver	19 (6.9)	20 (7.3)
Brain	36 (13.1)	41 (41.9)
PD‐L1 expression level^a^, *n* (%)		
<1%/indeterminate^c^	120 (43.6)	120 (43.6)
1%–49%	82 (29.8)	81 (29.5)
≥50%	73 (26.5)	74 (26.9)

Abbreviations: ECOG PS, Eastern Cooperative Oncology Group performance status; PD‐L1, programmed death‐ligand 1; TNM, the TNM classification of malignant tumors; UICC, Union for International Cancer Control.

^a^ Interactive Web Response System source.

^b^ Former smokers included anyone who had smoked even a small quantity of any tobacco and had stopped smoking at the time of screening. Current smokers are anyone who had a smoking habit of any tobacco regardless of smoking duration/quantity at the screening.

^c^ There were five patients with indeterminate PD‐L1 expression levels in both arms.

## 4 DISCUSSION

We performed updated analyses of treatment‐naïve patients with advanced nsNSCLC without driver mutations who were enrolled in a placebo‐controlled randomized trial in three Asian countries. At the data cutoff at which time the minimum follow‐up was 19.4 months, the nivolumab arm showed longer OS vs. placebo (HR 0.74, 95% CI 0.58–0.94). This compares with an HR of 0.85 in the primary analysis, which had a shorter minimum follow‐up (7.4 months). These findings expand on our prior analysis,^3^ demonstrating that the significant improvements in progression‐free survival and higher objective response in the nivolumab arm are coupled with a prolonged survival time compared with placebo.

To our knowledge, this is the first randomized, placebo‐controlled trial to reveal a survival benefit of an anti–PD‐1 antibody (nivolumab) administered together with bevacizumab and chemotherapy as initial, first‐line therapy of advanced nsNSCLC. An improvement in the tumor microenvironment may be conferred through synergistic effects of bevacizumab and nivolumab.^6^, ^7^ Although an updated analysis of PFS was not performed, PFS was also improved at the earlier cutoff.^3^ The median OS value of 30.8 months that was observed in the nivolumab arm is also the longest median OS published to date for all Phase III studies testing immune checkpoint inhibitors for treating nsNSCLC lacking driver mutations.^8^, ^9^, ^10^ We also note that the placebo arm's median OS (24.7 months) was numerically similar to or actually longer than that reported in other studies of PCB.^8^, ^11^, ^12^ In a Phase III study that enrolled wild‐type metastatic NSCLC patients, the authors reported that first‐line nivolumab combined with ipilimumab and chemotherapy prolonged OS in the Asian subpopulation relative to that observed in all randomized patients,^13^, ^14^ implying nivolumab combination therapy prolonged survival in Asian patients.

When patients were divided into subgroups by tumor PD‐L1 expression level, we observed a significant OS benefit in the PD‐L1 1%–49% group and a fair OS benefit in the <1%/indeterminate and ≥ 50% groups. This is inconsistent with the prior data showing that the PFS benefit was observed regardless of the PD‐L1 expression level, and that the PD‐L1 1%–49% group had the lowest numerical PFS benefit.^3^ Although PD‐L1 expression was a stratification factor, the possibility that some bias in patient background factors affected the prognosis cannot be ruled out.

The effects of first‐line atezolizumab combined with PCB for treating wild‐type metastatic nsNSCLC were recently reported in a Phase III study.^8^ In the intention‐to‐treat wild‐type analysis, the OS was significantly prolonged in the atezolizumab arm,^8^ for which the HR was 0.80, similar to the value in our study. However, in the Asian population, the HR for OS in the atezolizumab plus PCB arm was 1.18, although only 12% of patients in that study were Asian.^8^ Therefore, our study is, to the best of our knowledge, the first to reveal an add‐on survival effect of nivolumab when combined with PCB in Asian patients with wild‐type metastatic nsNSCLC.

The main limitation of this updated survival analysis is that only OS data were obtained; PFS and safety data were not updated. Further follow‐up of OS, PFS, and safety and future analyses are needed to comprehensively discuss the benefits of nivolumab.

In conclusion, nivolumab plus PCB provided superior OS vs. placebo plus PCB for the initial (first‐line) treatment of patients with metastatic nsNSCLC. Therefore, we believe that nivolumab plus PCB is a viable new option for the initial treatment of metastatic nsNSCLC without driver mutations in *ALK*, *EGFR*, or *ROS1*.

## AUTHOR CONTRIBUTIONS

**Hye Ryun Kim:** Conceptualization (lead); investigation (lead); methodology (lead); resources (lead); writing – original draft (equal); writing – review and editing (lead). **Shunichi Sugawara:** Investigation (equal); resources (equal); writing – review and editing (supporting). **Jong Seok Lee:** Investigation (supporting); resources (supporting); writing – review and editing (supporting). **Jin‐Hyoung Kang:** Investigation (supporting); resources (supporting); writing – review and editing (supporting). **Naoki Inui:** Investigation (supporting); resources (supporting); writing – review and editing (supporting). **Toyoaki Hida:** Investigation (supporting); resources (supporting); writing – review and editing (supporting). **Ki Hyeong Lee:** Investigation (supporting); resources (supporting); writing – review and editing (supporting). **Tatsuya Yoshida:** Investigation (supporting); resources (supporting); writing – review and editing (supporting). **Hiroshi Tanaka:** Investigation (supporting); resources (supporting); writing – review and editing (supporting). **Cheng‐Ta Yang:** Investigation (supporting); resources (supporting); writing – review and editing (supporting). **Makoto Nishio:** Conceptualization (equal); investigation (equal); methodology (equal); resources (equal); writing – review and editing (equal). **Yuichiro Ohe:** Conceptualization (equal); investigation (equal); methodology (equal); resources (equal); writing – review and editing (equal). **Tomohide Tamura:** Conceptualization (equal); investigation (equal); methodology (equal); resources (equal); writing – review and editing (equal). **Nobuyuki Yamamoto:** Conceptualization (supporting); investigation (equal); methodology (supporting); resources (supporting); writing – review and editing (supporting). **Chong‐Jen Yu:** Conceptualization (supporting); investigation (supporting); methodology (supporting); resources (supporting); writing – review and editing (supporting). **Hiroaki Akamatsu:** Conceptualization (supporting); investigation (equal); methodology (supporting); resources (equal); writing – review and editing (supporting). **Shigeru Takahashi:** Conceptualization (equal); data curation (lead); formal analysis (lead); methodology (equal); writing – original draft (supporting); writing – review and editing (equal). **Kazuhiko Nakagawa:** Conceptualization (lead); investigation (lead); methodology (lead); project administration (lead); resources (lead); supervision (lead); writing – original draft (lead); writing – review and editing (lead).

## FUNDING INFORMATION

This study was funded by Ono Pharmaceutical Co., Ltd., and Bristol‐Myers Squibb.

## CONFLICT OF INTEREST STATEMENT

Ono Pharmaceutical Co., Ltd. and Bristol‐Myers Squibb funded the study, provided the study drugs, and were involved in the collection, analysis, and interpretation of the data. Employees of the sponsors reviewed and commented on the manuscript. The authors had full access to the data and took final responsibility for the decision to publish the manuscript.

The authors report the following disclosures; remuneration of 1 million yen or more: S.T. (Ono Pharmaceutical); lecture fees/honoraria/other fees of 500,000 yen or more: H.‐R.K. (Ono Pharmaceutical, AstraZeneca, Roche, Boehringer Ingelheim), S.S. (Ono Pharmaceutical, Bristol‐Myers Squibb), T.H. (Ono Pharmaceutical, Bristol‐Myers Squibb), K.‐H.L. (Bristol‐Myers Squibb, MSD, AstraZeneca, Pfizer, and Eli Lilly), T.Y. (Ono Pharmaceutical, Chugai, AstraZeneca), H.T. (AstraZeneca, Chugai), C.‐T.Y. (Ono Pharmaceutical, Boehringer Ingelheim, AstraZeneca, Chugai, Pfizer, Takeda, Novartis, Roche, Eli Lilly, Merck, MSD), M.N. (Ono Pharmaceutical, Bristol‐Myers Squibb), Y.O. (AstraZeneca, Chugai), T.T. (Cmic ShiftZero), H.A. (AstraZeneca), N.Y. (MSD, AstraZeneca, Chugai, Eli Lilly, Boehringer‐Ingelheim, Pfizer, Ono Pharmaceutical, Takeda), K.N. (Eli Lilly, Chugai, Ono Pharmaceutical); annual contract research grants of 1 million yen or more: H.‐R.K (Ono Pharmaceutical), S.S. (Ono Pharmaceutical, Bristol‐Myers Squibb), J.‐S.L. (Ono Pharmaceutical), J.‐H.K. (Ono Pharmaceutical), N.I. (Ono Pharmaceutical), T.H. (Ono Pharmaceutical, Bristol‐Myers Squibb), K.‐H.L. (Ono Pharmaceutical), T.Y. (Ono Pharmaceutical, Chugai, AstraZeneca, Amgen, Novartis, Bristol‐Myers Squibb, Takeda), H.T. (Ono Pharmaceutical), C.‐T.Y. (Ono Pharmaceutical), M.N. (Ono Pharmaceutical, Bristol‐Myers Squibb), Y.O. (AstraZeneca, Chugai, Eli Lilly, Ono Pharmaceutical, Bristol‐Myers Squibb, Kirin, Dainippon‐Sumitomo, Pfizer, Taiho, Novartis, Takeda, Kissei, Daiichi‐Sankyo, Janssen, LOXO), N.Y. (Chugai, Toppan printing, Cmic ShiftZero, MSD, Pfizer, PPD, IQVIA, Eli Lilly, A2 healthcare, Boehringer Ingelheim, Terumo, Takeda, AstraZeneca, Amgen, Jansen, Taiho, Ono Pharmaceutical), C.‐J.Y. (Ono Pharmaceutical), H.A. (Chugai, Amgen), K.N. (Ono Pharmaceutical, MSD, Daiichi Sankyo, Taiho, Chugai, SYNEOS HEALTH CLINICAL, Japan Clinical Research Operations, AstraZeneca, IQVIA Services JAPAN, Covance Japan, Takeda, GlaxoSmithKline, Sanofi, EPS Corporation, Novartis, Medical Research Support); scholarship/endowments of 1 million yen or more: N.Y. (Taiho, Chugai, Daiichi‐Sankyo, Ono Pharmaceutical), K.N. (Takeda, Chugai, Ono Pharmaceutical). Y.O. is a member of the editorial board of *Cancer Science*.

## ETHICS STATEMENT

The study was approved by the Institutional Review Board or Independent Ethics Committee at all participating sites. All patients provided informed consent to participate in this study. The study was registered on ClinicalTrials.gov (NCT03117049) and Japan Pharmaceutical Information Center (JapicCTI‐173560).

## Data Availability

Qualified researchers may request Ono Pharmaceutical Co., Ltd. to disclose individual patient‐level data from clinical studies through the following website: https://www.clinicalstudydatarequest.com/. For more information on the policy of Ono Pharmaceutical Co., Ltd. for the Disclosure of Clinical Study Data, please visit https://www.ono.co.jp/eng/rd/policy.html.
